# Electrochemical detection of diclofenac and clindamycin using ZnO nanorods/RGO nanocomposite modified electrode

**DOI:** 10.1039/d5ra07011b

**Published:** 2025-12-10

**Authors:** Gokul Sridharan, Surendar Balu, Raji Atchudan, Chandramohan Govindasamy, Dhanraj Ganapathy, Sandeep Arya, Ashok K. Sundramoorthy

**Affiliations:** a Centre for Nano-Biosensors, Department of Prosthodontics, Saveetha Dental College and Hospitals, Saveetha Institute of Medical and Technical Sciences Poonamallee High Road, Velappanchavadi Chennai 600077 Tamil Nadu India ashok.sundramoorthy@gmail.com; b School of Chemical Engineering, Yeungnam University Gyeongsan 38541 Republic of Korea; c Department of Community Health Sciences, College of Applied Medical Sciences, King Saud University P.O. Box 10219 Riyadh 11433 Saudi Arabia; d Department of Physics, University of Jammu Jammu Jammu and Kashmir 180006 India

## Abstract

A new electrochemical sensor based on electrochemically reduced graphene oxide (RGO) and zinc oxide (ZnO) nanorods was developed for the determination of the nonsteroidal anti-inflammatory drug (Diclofenac) and antibiotic drug (Clindamycin) for applications in human health monitoring. Graphene oxide (GO) and ZnO nanorods were synthesised by Hummer's and hydrothermal methods, respectively. The ZnO/GO composite (1 : 1 ratio) was prepared using the sonochemical method. As-prepared GO, ZnO, and ZnO/GO nanocomposite materials are characterised by UV-vis spectroscopy, Fourier transform infrared spectroscopy (FTIR), X-ray diffraction analysis (XRD), high-resolution scanning electron microscopy (HR-SEM), Energy dispersive spectroscopy (EDAX), Thermogravimetric analysis (TGA), Zeta potential/Dynamic light scattering (DLS), and Electrochemical impedance spectroscopy (EIS). In addition, the ZnO/GO nanocomposite film-coated electrode was reduced electrochemically to ZnO/RGO. The electrochemical reaction of the ZnO/RGO was investigated by using cyclic voltammetry (CV). The ZnO/RGO/GCE-based electrochemical sensor showed the lowest detection limits for diclofenac (DCF) and clindamycin (CMC) as 0.079 µM and 0.018 µM, respectively. The sensitivity of the sensor was 0.127 µA µM^−1^ cm^−2^ for DCF and 0.153 µA µM^−1^ cm^−2^ for CMC, and a linear response in the range of 0.5 to 85.0 µM for DCF and 0.05 to 36.50 µM for CMC was observed. The ZnO/RGO/GCE sensor was tested in a real sample of human urine and found a recovery range of 90.0% to 106.0%. Overall, the proposed dual electrochemical sensor can be used in real-world applications.

## Abstract 2

A new electrochemical sensor based on electrochemically reduced graphene oxide (RGO) and zinc oxide (ZnO) nanorods was developed for the determination of the nonsteroidal anti-inflammatory drug (Diclofenac) and antibiotic drug (Clindamycin).

## Graphical abstract

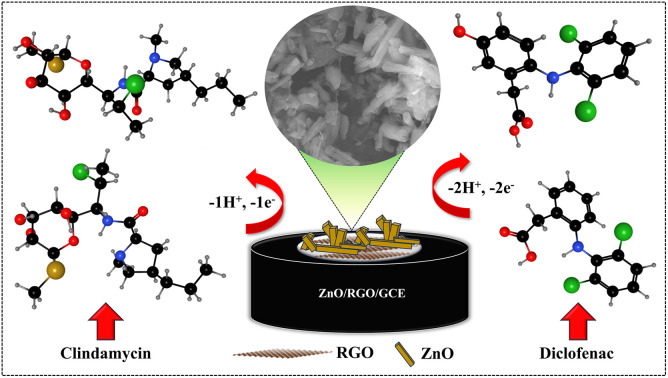

## 1. Introduction

Clindamycin (CMC) belongs to the lincosamide class of antibiotics and is frequently prescribed for the treatment of various bacterial infections. It is effective against a range of conditions, including vaginal infections, staphylococcal and streptococcal infections, as well as malaria. In addition, CMC is utilised to combat bacterial invasions affecting the lungs, blood, skin, and internal organs.^1,2^ CMC is quickly absorbed when used orally, with 90% absorption being considered excellent. The highest concentration is reached in around 60 minutes when taken orally.^3^ Oral CMC dose recommendations are 150–300 mg every 6 hours for moderately severe infections and 300–450 mg every 6 hours for severe infections.^4^ Despite its effectiveness, it can induce skin dryness, peeling, nausea, and, in rare cases, serious disorders such as leukopenia and Stevens-Johnson syndrome.^5,6^ Further, there is a chance to build up resistance with continuous usage of this drug. Diclofenac (DCF) is a nonsteroidal anti-inflammatory drug (NSAID) that acts by blocking the cyclooxygenase enzyme, thereby reducing the formation of prostaglandins, which cause inflammation, discomfort, and fever. Since its release in 1973, DCF has been frequently used for pain, cartilage damage, swelling, and chronic illnesses such as rheumatoid arthritis (RA) and osteoarthritis.^7,8^ Because of its limited solubility, the medicine is frequently administered as a sodium salt and can be acquired in various forms, such as oral pills, capsules, regional gels, and injectable solutions.^9^ Nowadays, the combined usage of multiple therapeutic drugs (polytherapy or combination therapy) is quite common for treating complex and chronic conditions.^10,11^ However, using CMC and DCF together can increase the risk of serious side effects, particularly a rare but life-threatening complication like colon perforation with acute peritonitis, especially after dental surgery.^12^ Hence, it is highly necessary to have dual-drug detection sensors for regular diagnosis. Accordingly, in our previous research, we have reported a similar dual-drug-based sensor for the detection of paracetamol (NSAID) and amoxicillin (antibiotic) using Copper oxide/graphitic carbon nitride-based nanocomposite as an electrocatalyst.^13^ For the sensing of CMC and DCF there are several detection techniques have been established, including high-performance liquid chromatography (HPLC),^14,15^ spectrophotometry,^16,17^ capillary electrophoresis,^18,19^ and chemiluminescence.^20^ Electrochemical (EC) methods have become valuable alternatives, due to their high sensitivity, rapid analysis, cost-effectiveness, precise detection of analytes, quick response times, and simple instrumentation for human health monitoring. These approaches perform well even in complex sensing systems and can be miniaturised, making them ideal for portable and point-of-care applications.^21–23^ Last few years, the EC detection of DCF has been highly active, especially by using various carbon-based electrode materials. For example, Sundaresan *et al.*^24^ developed a sensor using exfoliated graphite-supported cobalt ferrite nanocomposite, which exhibits a remarkable sensitivity. Recently, Măghinici *et al.*^25^ proposed a DCF sensor with a lower LOD using phenanthroline immobilised graphene oxide-based electrode. El-Wekil *et al.*^26^ developed an effective simultaneous sensor for DCF and esomeprazole using an RGO-coated carbon paste electrode. In contrast, for the EC detection of CMC, there are very limited studies that have been published. Recently, Ahmad *et al.*^27^ proposed CMC sensors by using a Zn–Al layered double hydroxide (LDH) nanoparticle-based electrode, which showed a wide range of detection in human urine and serum samples. However, there are still research gaps in the electrochemical sensing of DCF and CMC with the lowest limit of detection, a wide linear range, the lowest oxidation potential, high selectivity, and stability. Thus, we have attempted to prepare a facile and effective nanocomposite, which can be able to detect both DCF and CMC. Compared to all other catalytic materials, graphene is still a highly interesting and outstanding material. Over the past few decades, graphene-based composites have garnered significant interest in EC applications due to graphene's outstanding electrical conductivity, mechanical strength, and high surface area.^28–30^ However, graphene sheets tend to restack through π–π interactions, which limits their effective surface area and practical performance. This limitation can be mitigated by incorporating inorganic nanostructures into the graphene matrix, which not only prevents restacking but also enhances its EC functionality.^31^ For sensor applications, graphene is functionalised so that it can be well dispersed in water or in other polar solvents.^32^ However, graphene oxide (GO) exhibits electrical insulating properties; upon removal of oxygen groups, electron conductivity is significantly enhanced through the partially restored sp^2^-bonded carbon network with aromatic graphitic domains.^33^

Zinc oxide (ZnO) is considered one of the most promising metal oxides for advanced sensing applications among a variety of options, especially when combined with reduced graphene oxide (RGO).^34^ Compared to many other metal oxides, its broad bandgap (3.37 eV), rich surface defect chemistry, and 60 M eV exciton binding energy allow for rapid charge transport and improved analyte adsorption, resulting in higher sensitivity. Additionally, ZnO exhibits chemical stability, biocompatibility and can be synthesised in various nanostructures through scalable and cost-effective methods.^35^ Compared to other configurations, ZnO nanorods demonstrated enhanced electron transport capabilities, along with superior mechanical strength and durability.^36^ The integration of ZnO nanorods into reduced graphene oxide (RGO) results in a synergistic composite with superior electrical conductivity, structural stability, and enhanced electrocatalytic activity.

**Scheme 1:**
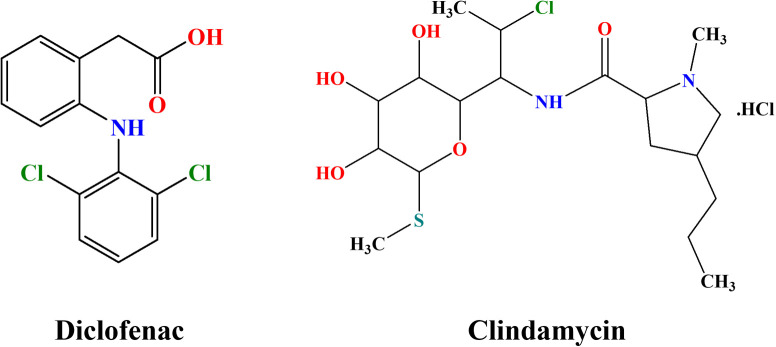
Chemical structures of diclofenac and clindamycin.

The ZnO/RGO nanocomposite (NC) has demonstrated markedly improved EC performance compared to its individual components, making it a promising material for sensor and energy-related applications.^37–39^ It has been documented that the addition of ZnO and RGO has synergistic effects. ZnO interacts with the analyte as an activator, while RGO increases ZnO's EC reaction efficiency.^40^ Moreover, RGO promotes high electron transit while inhibiting charge carrier recombination, which improves composite efficiency.^41^ Consequently, there can be a notable enhancement in the ZnO/RGO composite's electrocatalytic activity. In our previous research, we have used ZnO/GO as an efficient catalyst for the enhanced detection of gallic acid.^42^ To the best of the authors' knowledge, no prior studies have been published on the use of ZnO/RGO NC for the determination of DCF and CMC. Therefore, this study aimed to develop a ZnO/RGO/GCE for the quantitative detection of DCF and CMC. The sonochemical method was used to prepare the ZnO/RGO NC, whereas modified Hummer's technique was used to synthesise graphene oxide (GO), and the hydrothermal method was used to synthesise ZnO nanorods. Using cyclic voltammetry and amperometry methods, the electrocatalytic performance of the NC was investigated to assess its potential use as an EC sensor for CMC and DCF detection (Scheme 1).

## 2. Experimental

### 2.1. Chemicals and instruments

Zinc acetate dihydrate, hydrazine hydrate, sodium nitrate (NaNO_3_), sulfuric acid (H_2_SO_4_), and hydrogen peroxide (H_2_O_2_) were purchased from Sisco Research Laboratories Pvt, Ltd (SRL), India. Graphite flakes (+50 mesh particle size) were purchased from Sigma Aldrich Chemicals Pvt. Ltd. Clindamycin hydrochloride (Himedia Laboratories Pvt. Ltd., India) and diclofenac sodium salt (Tokyo Chemical Industry (India) Pvt. Ltd) were used for analysis. The studies were conducted in a 0.1 M phosphate buffer solution (PBS, pH 7.4) that was prepared using sodium dihydrogen phosphate monohydrate (H_2_NaPO_4_·H_2_O) and sodium phosphate dibasic heptahydrate (Na_2_HPO_4_·7H_2_O). We purchased these chemicals from Spectrochem Pvt., Ltd in India. All of the remaining reagents were of analytical grade and were used without further purification. All experiments were conducted using DI H_2_O.

### 2.2. Synthesis of graphene oxide

Graphite flakes (1 g) are mixed with 1 g of NaNO_3_. This mixture is then dispersed in 50 mL of conc. H_2_SO_4_. The solution is stirred for 40 minutes before being transferred to an ice bath to maintain a temperature of 40–50 °C. Next, 8 grams of KMnO_4_ is slowly added until the colour changes to purple green, with stirring continued for 4 hours. Subsequently, H_2_O_2_ (6 mL) is added to produce a brown solution. To this final suspension, 80 mL of distilled water (DI H_2_O) is added. The mixture is then centrifuged several times with DI H_2_O to reach a neutral pH. Finally, the resultant product is dried at 90 °C for 24 hours.^43,44^

### 2.3. Preparation of GO/ZnO nanocomposite

Rod-shaped ZnO was synthesised using the hydrothermal method, starting by dissolving 2.18 g of zinc acetate dihydrate (Zn(CHCOO)_2_·2H_2_O) in DI H_2_O (60 mL), ensuring complete dissolution through rapid stirring. In this reaction, hydrazine hydrate (4 mL) is added dropwise to the zinc acetate dihydrate solution. The mixture was placed in a Teflon-lined reaction kettle, and a hydrothermal reaction at 155 °C for 24 h was carried out. After the reaction, the resulting white precipitate was centrifuged at 5000 RPM for 5 minutes, with DI H_2_O and ethanol multiple times. Finally, the ZnO samples were obtained through freeze-drying at −80 °C. After that, 5 mg of each GO and ZnO (1 : 1) were dispersed in DI H_2_O and probe-sonicated for 2 h at 30% amplitude (3 Sec on, 2 Sec off). The resulting mixture was used for further characterisation and sensor applications.

### 2.4. Characterisation

Using the UV-Jasco spectrophotometer, the UV-vis spectrum of ZnO/GO was acquired. The surface morphology of the nanocomposite was then analysed using a High-Resolution Scanning Electron Microscope (HR-SEM). After being drop cast onto aluminium foil, the ZnO/GO nanocomposite was cured on a hot plate, stored for a full night at 100 °C in a hot air oven, and then examined using an HR-SEM operating at 30 kV (QUANTA 200F SAIF, IITM). A Fourier Transform Infrared (FTIR) spectrophotometer (ALPHA II, Bruker, Germany) was used to determine the functional groups of the nanocomposite. The crystalline structure of the ZnO/GO was confirmed by obtaining an X-ray diffraction (XRD) pattern from 5° to 80° using an X-ray diffractometer (D8-Advance, BRUKER, Mannheim, Germany). With 1.5406 Å wavelength Cu Kα radiations, the XRD instrument was run at 40 kV voltage and 15 mA current. A 10° min^−1^ scan rate was used to acquire the XRD pattern. Surface charge and average hydrodynamic size of the ZnO/GO NC were analysed using a zeta potential analyser and a Dynamic Light Scattering (DLS) analyser (Adv series-ultra, Malvern Panalytical, Worcestershire, UK). Thermal stability of the ZnO/GO NC was tested using a thermogravimetric analyser (TGA) 8000 (PerkinElmer, Shelton, Connecticut).

### 2.5. Electrochemical measurements and sensor fabrication

An EC workstation CHI 760E (CH Instruments, Austin, TX, USA) was used for all EC measurements. The reference electrode in a standard three-electrode setup was Ag/AgCl maintained in 1 M KCl. ZnO/GO, subsequently electrochemically reduced to ZnO/RGO, was coated on a Glassy Carbon Electrode (GCE) that served as the working electrode. Previously, GCE was polished with 0.05 µm alumina slurry and bath sonicated in DI H_2_O to create a surface that resembled a mirror. After that, GCE underwent ten cycles of potential scanning (0 to 1.0 V) in 0.1 M H_2_SO_4_ for EC treatment. Following surface treatment, 7 µL of ZnO/GO dispersion was dropped over the GCE and subsequently dried at 50 °C. The electrode surface was cleaned of the unbound nanoparticles by immersing ZnO/GO/GCE in 10 mL of DI H_2_O and dried at room temperature for a few minutes. To prepare a ZnO/RGO/GCE, ZnO/GO/GCE was electrochemically reduced in 0.1 M PBS (pH 7.4) from 0 to −1.0 V for 10 CV cycles continuously at a scan rate of 50 mV s^−1^.

### 2.6. Sample preparation

DCF and CMC stock solutions (10 mM) were prepared by dissolving 31.8 mg and 42.5 mg in 0.1 M PBS of 10 mL, respectively, then diluted to 1 mM and 0.1 mM for amperometric analysis. All stocks were stored at 4 °C in the dark. DCF and CMC concentrations were determined using a ZnO/RGO/GCE sensor *via* the standard addition method and real sample analyses. In order to detect DCF and CMC in real-world samples using the proposed ZnO/RGO/GCE sensor, human urine was taken from a healthy adult after obtaining informed consent for all procedures involving human subjects. The sample was first diluted tenfold with 0.1 M PBS, pH 7.4, then spiked with 1 mM standard solutions of DCF and CMC. Before spiking, the urine was filtered using Whatman No. 1 filter paper. The prepared sample was stored at 4 °C for later analysis.

## 3. Results and discussion

### 3.1. UV-visible spectroscopy

**Fig. 1:**
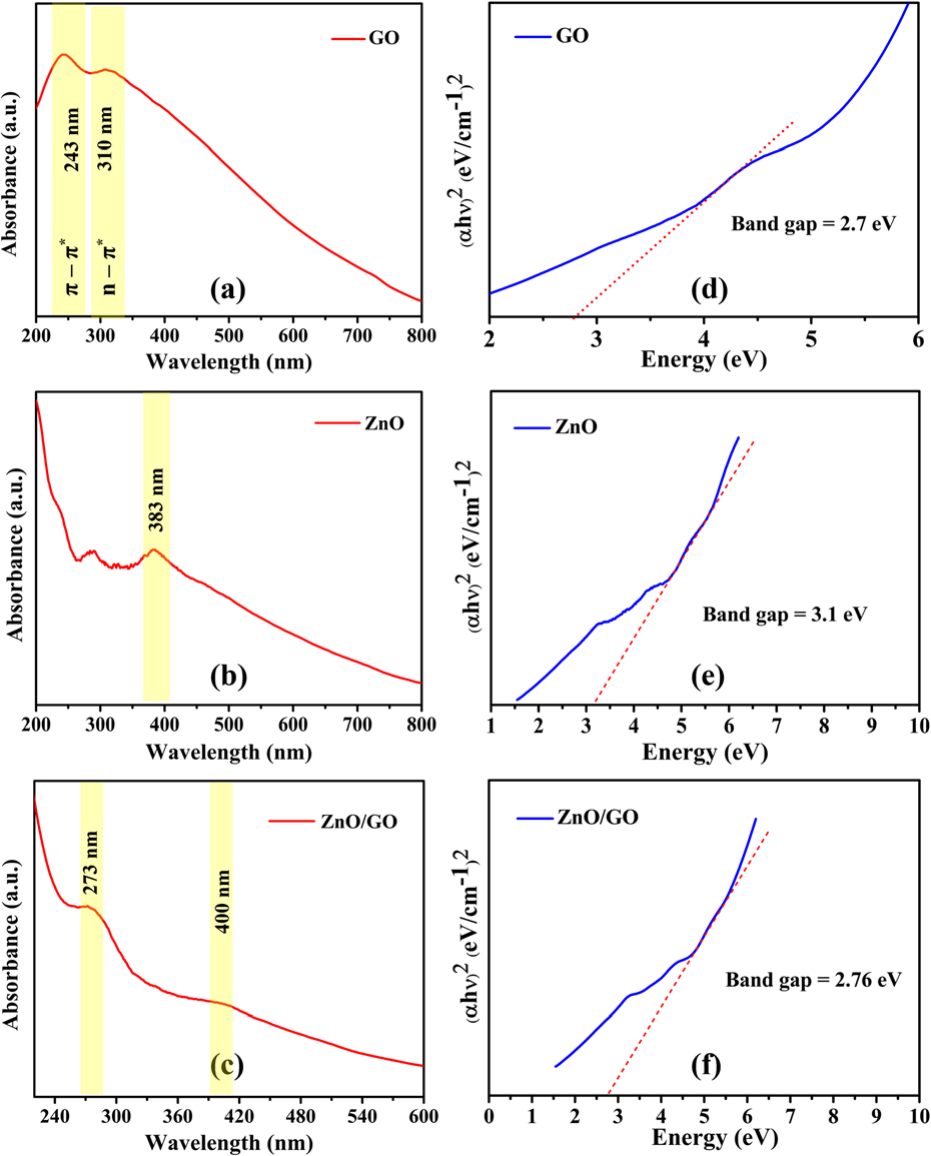
UV-visible spectra of (a) GO, (b) ZnO, and (c) ZnO/GO. Tauc plots of (d) GO, (e) ZnO, and (f) ZnO/GO film.

The UV-visible spectroscopy was employed to investigate the optical properties of the synthesised materials, which included GO, ZnO, and ZnO/GO NC. The UV-vis absorbance spectrum of GO is shown in Fig. 1a, which shows a clear absorption peak at 243 nm. This peak represents the π–π* transitions associated with the conjugated C=C bonds found in the aromatic rings of the GO structure.^45^ In addition, a shoulder peak approximately 310 nm might be observed due to the n–π* transitions of the C=O bonds. This implies that oxygen-containing functional groups, including carboxyl and epoxy, are present on the GO surface and change its electrical structure by interfering with conjugation between graphene sheets.^46^ These functional groups are required to improve the material's chemical reactivity and interaction with other materials, such as ZnO, in composite structures. The UV-vis spectrum of ZnO nanoparticles is shown in Fig. 1b. ZnO has a strong absorption edge at around 383 nm, aligning with its related band gap. This absorption results from electrical transitions from the valence band to the conduction band, typical of a wide-band gap semiconductor such as ZnO.^46,47^ The high crystallinity and low defect content of the ZnO nanoparticles are shown by the sharp absorption edge. The detected absorption in the UV spectrum indicates that ZnO is a proficient absorber of UV light, rendering it appropriate for applications including UV filtration and photocatalysis. Fig. 1c shows the UV-visible spectrum of the ZnO/GO NC, indicating a wider absorption spectrum relative to the individual components. The ZnO/GO NC has an absorption edge approximately at 273 nm and 400 nm, which are slightly red-shifted relative to the GO and pure ZnO, respectively. This alteration indicates significant interactions between ZnO and GO, where GO's presence alters the electrical structure of ZnO. The ZnO/GO NC exhibits increased absorbance in the visible spectrum from 400 nm to 600 nm, a characteristic not seen in either pure ZnO or GO. The heightened absorbance in the visible spectrum is probably attributable to the charge transfer interactions between ZnO and GO. The optical band gaps of GO, ZnO, and the ZnO/GO NC were determined using Tauc plots to provide more insight into the electronic structure of these materials^48,49^ (Fig. 1d–f). Fig. 1d shows the Tauc curve for GO, indicating an optical band gap of roughly 2.7 eV. The reduced band gap is attributable to the oxygen functional groups on the GO surface, which provide localised electronic states and decrease the energy necessary for electronic transitions. Graphene oxide can act as either a semiconductor or an insulator, depending on the level of oxidation,^50^ and its band gap enables modest absorption in the visible spectrum, a property that is augmented when combined with composite systems like ZnO/GO.^42^Fig. 1e shows the Tauc plot for ZnO, indicating a band gap of 3.1 eV, aligning with the intrinsic band gap of pure ZnO.^49,51^ The wide band gap signifies ZnO's robust UV absorption properties, rendering it appropriate for applications involving UV light processing. The band gap of the ZnO/GO NC, as shown in Fig. 1f, is notably less than that of pure ZnO at around 2.76 eV. The synergistic interaction between ZnO and GO is the cause of a decrease in the band gap. When GO is integrated into the ZnO structure, a heterojunction is created that promotes charge transfer and lowers electron–hole pair recombination. Because it enables the composite to use a larger range of the solar spectrum, this improved charge separation is essential for increasing the effectiveness of the electronic transition. The composite's ability to absorb more visible light and perform better in energy-related applications is indicated by the smaller band gap, which also suggests a change in the composite's optical characteristics. Due to the presence of GO in the nanocomposite, there are more opportunities for increased electronic interactions, which lowers the band gap overall and changes the material's optical characteristics.

### 3.2. FT-IR Spectroscopic analysis

**Fig. 2:**
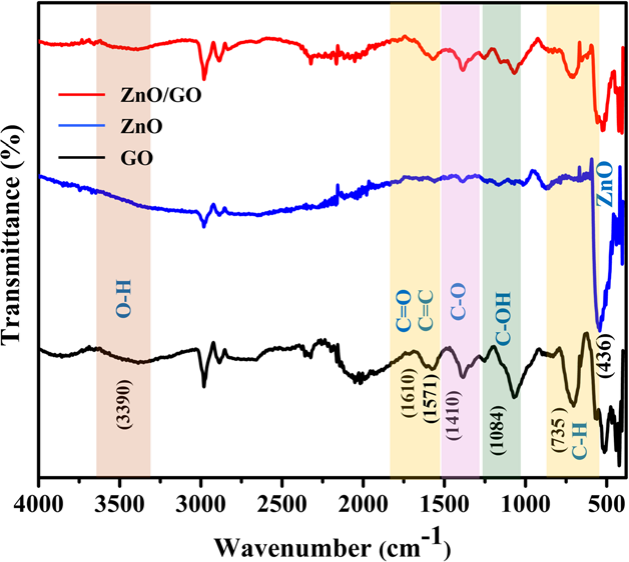
FTIR spectra of GO (black), ZnO (blue), and ZnO/GO film (red).

The structural clarification and examination of the functional groups in the materials were carried out using FTIR spectra analysis (Fig. 2). Because of the stretching and bending vibrations of the –OH groups, GO displayed a large peak between 3100 and 3600 cm^−1^. It concludes that the sample is very hydrophilic and that water molecules have been adsorbed on GO. The 735 cm^−1^, 1084 cm^−1^, 1410 cm^−1^, 1571 cm^−1^, and 1610 cm^−1^, peaks revealed the functional groups of C–H, C–OH, C–O, C=C, and C=O, respectively.^52,53^ The oxidation process used in Hummer's approach produces these oxygen functional groups.

However, even after oxidation, graphite's primary structure will remain, as seen by the 1571 cm^−1^ peak, which shows that the alkene's C=C is preserved (black curve).^54^ The ZnO frequencies obtained from the IR spectra reported in the literature.^38^ A fingerprint absorption area below 1000 cm^−1^ is produced by the metal-oxide nanoparticle's interatomic vibrations.^55^ The 436 cm^−1^ peak indicates the deformation vibration of ZnO (blue curve). Furthermore, the reduction of intensity at 3390 cm^−1^ (O–H), 735 cm^−1^ (C–H), and 436 cm^−1^ (ZnO) in the FTIR spectrum (red curve) shows the composition of ZnO/GO.

### 3.3. XRD and HR-SEM analysis

**Fig. 3:**
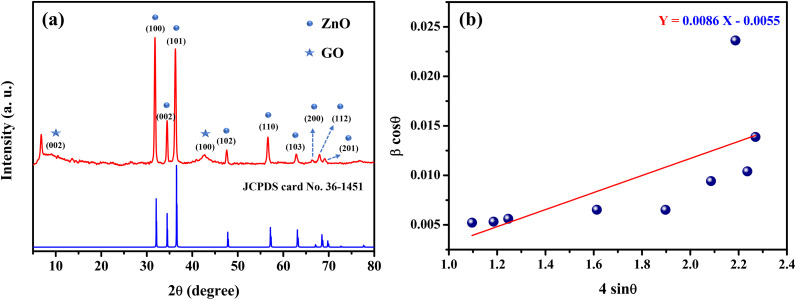
(a) X-ray diffraction (XRD) pattern of ZnO/GO composite with JCPDS data and (b) Williamson Hall (WH) plot.

Fig. 3a displays the XRD spectrum of ZnO/GO composite. XRD analyses validate that the diffraction pattern delineates the hexagonal wurtzite crystal structure of ZnO and the presence of GO. The orientation of GO is shown as (002) and (100) by the 2*θ* values at 8.91 and 42.77°, respectively. The ZnO crystallite's 2*θ* values at 31.80, 34.46, 36.29, 47.55, 56.62, 62.84, 66.28, 67.97, and 69.14° corresponding orientations were determined to be (100), (002), (101), (102), (110), (103), (200), (112), and (201) (JCPDS No: 36-1451).^56^ By applying the Scherrer (eqn (1)) and Williamson–Hall (WH) plot (eqn (2)) methods, the average crystallite size of ZnO was determined to be 18.98 nm and 26.33 nm, respectively.1
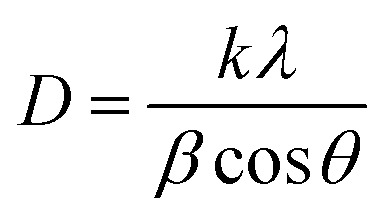
2
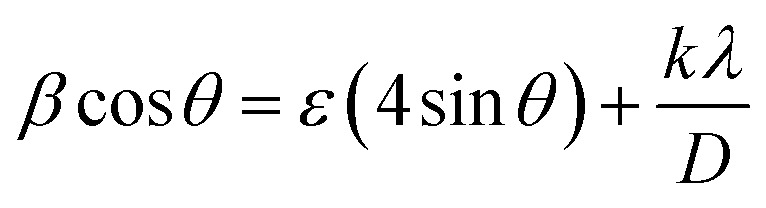


Eqn (2) denotes the Uniform Deformation Model (UDM), which assumes that strain is constant along all crystallographic axes.^57,58^*K* represents a dimensionless form factor (0.94), *λ* denotes the wavelength of Cu Kα’s, *D* is the average crystallite size, (*θ*) is Bragg's angle, *β* is the full-width half-maximum (FWHM), and strain (*ε*) is expressed in nanometres. Due to instrumentation errors and other factors, such as the micro-strains of the crystallites, the size of the crystallite in the WH plot has decreased.^59^ The WH graph's (Fig. 3b) slope value (0.0086), or 0.86%, was taken into consideration as the ZnO micro strain value. The positive value suggests the amount of micro strain is being influenced by tensile stress. Overall, XRD revealed the crystallite size, structure, and micro-strain.^60^

For composites to be utilised as electrocatalysts in EC sensors, surface morphology is the primary consideration.^61^

**Fig. 4:**
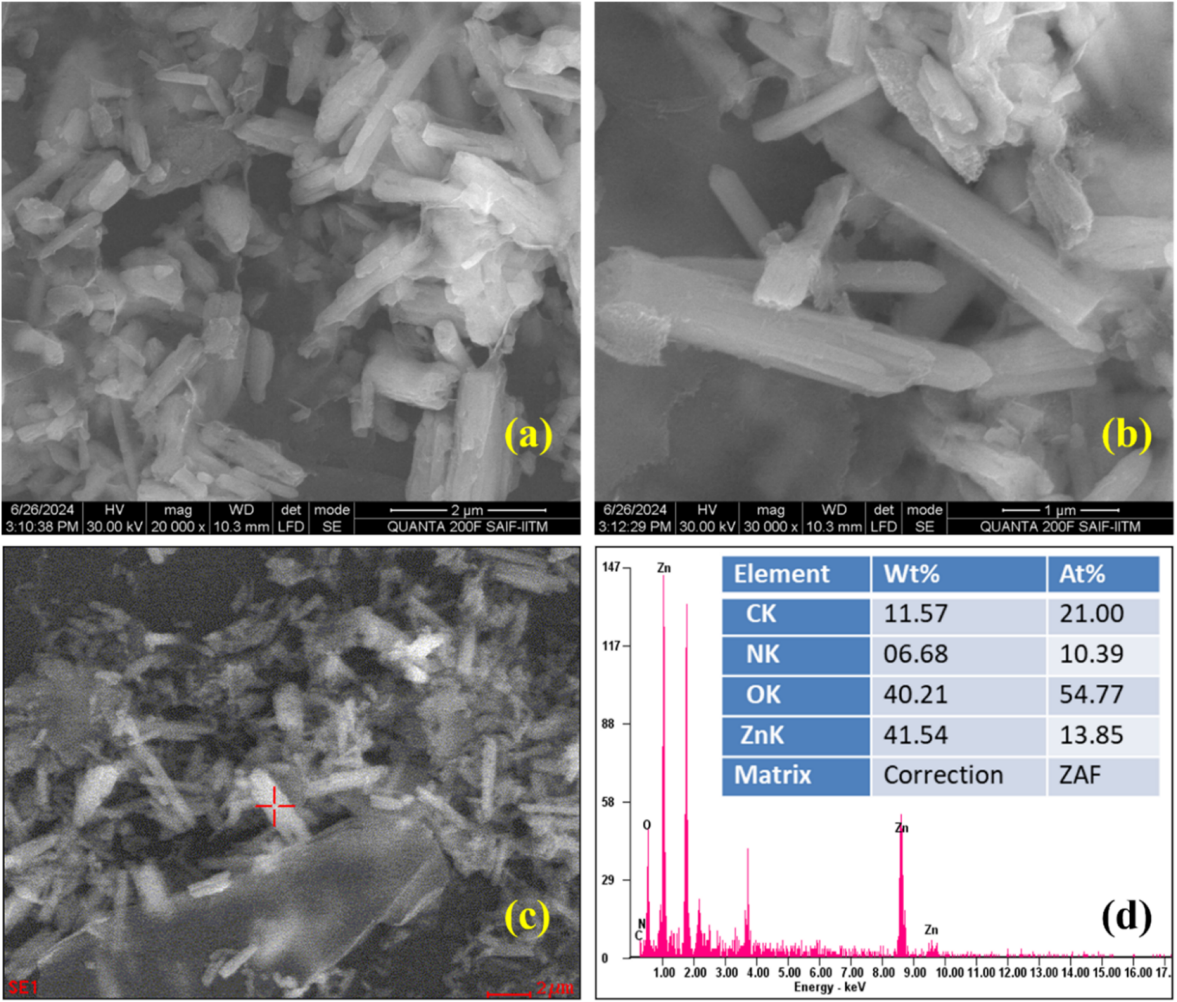
HR-SEM images of ZnO/GO composite under different magnifications (2 µm resolution (a) and 1 µm resolution (b)). HR-SEM image shows the scanned area (c) and the corresponding EDAX analysis (d).

ZnO coated with a thin layer of GO exhibits a rod-like morphology, as depicted in Fig. 4a at a magnification of 20 000× and resolution of 2 µm. According to Zhou *et al.*^62^ the formation of ZnO nanorods results from the influence of hydrazine hydrate during synthesis. An image at 30 000× magnification and 1 µm resolution is presented in Fig. 4b. The designated area for Energy Dispersive Spectroscopy (EDS) analysis is illustrated in Fig. 4c. Elemental composition of the nanocomposite (Fig. 4d) revealed percentages of 11.57% carbon, 6.68% nitrogen, 40.21% oxygen, and 41.54% zinc.

### 3.4. Thermal stability analysis

The TGA was employed to study the thermal stability of ZnO/GO NC, as presented in Fig. S1. The TGA curve reveals a four-step weight loss process, which was previously reported by Bekele *et al.*^63^ The initial phase, spanning 30–153 °C, exhibits a 5.05% reduction attributed to moisture evaporation. The second phase, between 152–300 °C, demonstrates a further 5.17% weight decrease, primarily due to the removal of functional groups that contain oxygen and partial decomposition of the carbon matrix. In the third phase, from 300–530 °C, a 4.67% loss is observed, resulting from the combustion of functional groups present in the GO and crystallisation of the ZnO nanorods^64^ in an air atmosphere. Finally, the fourth phase, extending from 530 to 833 °C, leads to a 9.91% decline in mass, which might correspond to the further degradation and recrystallisation of the NCs. However, the overall weight loss of the composite is only 24.8%, which shows that the ZnO/GO NC has high thermal stability.

### 3.5. DLS and zeta potential analysis

The hydrodynamic size of ZnO/GO NC was assessed by dynamic light scattering (DLS), yielding an average diameter of 812.8 nm, as shown in Fig. S2a. The size distribution analysis indicated a polydispersity index (PDI) of 0.594, suggesting a relatively uniform particle size distribution. Additionally, the average size of ZnO/GO NCs is smaller, and it's due to the ultra-dilution of the sample, which was corroborated by HR-SEM image (Fig. 4a) and aligns with the DLS findings. The zeta potential analyser was used to evaluate the surface charge and dispersion stability of the ZnO/GO; a larger positive or negative value denotes more stability of the nanoparticle dispersion. From Fig. S2b, the zeta potential distribution curve revealed that the surface charge of the ZnO/GO NCs was −26.24 mV, which reflects the presence of oxygenated functional groups on the surface of GO. In a neutral aqueous medium, ZnO typically exhibits a positive surface charge.^65^ However, the observed negative zeta potential is attributed to the presence of GO.

### 3.6. Electrocatalytic properties of ZnO/RGO composite

**Fig. 5:**
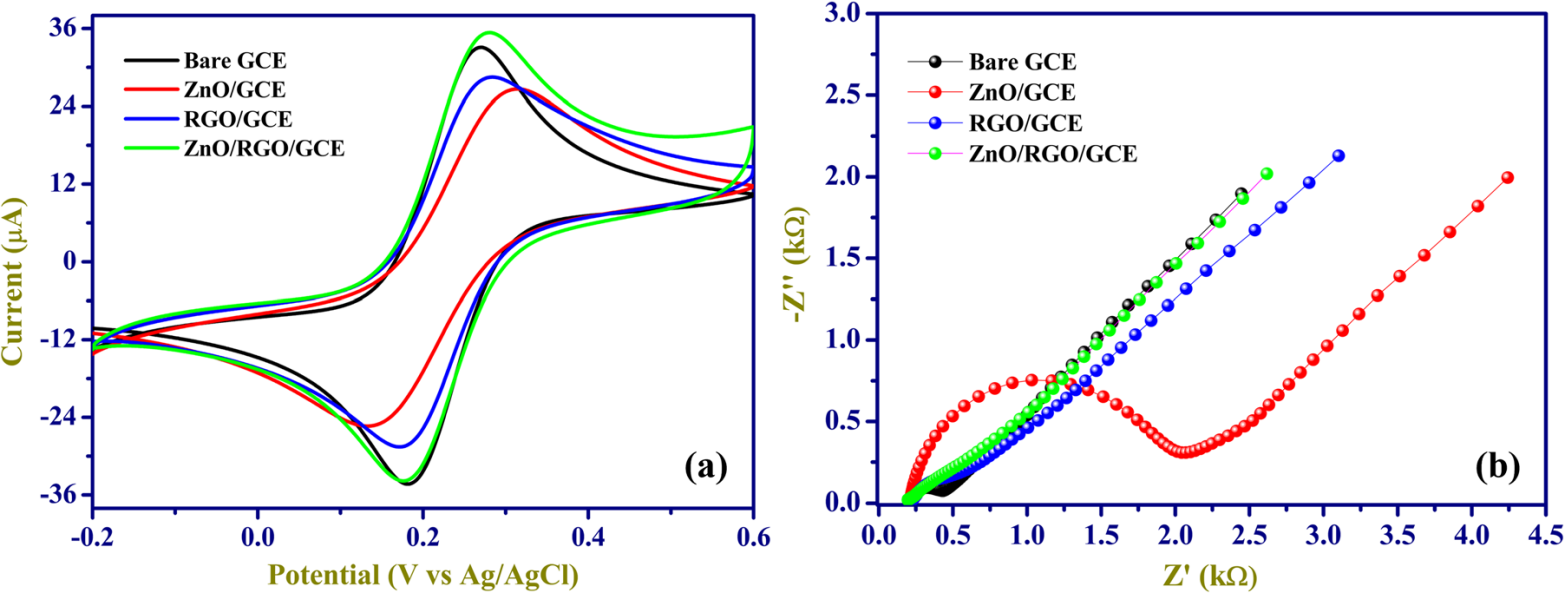
CVs (a) and EIS (b) of bare GCE (black), ZnO/GCE (red), RGO/GCE (blue), and ZnO/RGO/GCE (green) in 0.1 M KCl containing 2 mM [Fe(CN)_6_]^3−/4−^ (inset: Randles equivalent circuit).

Electrochemical impedance spectroscopy (EIS) investigations provided valuable insights into electron-transfer kinetics at the interface of the electrode–electrolyte on a working electrode.^66^Fig. 5a indicates the CVs of a bare GCE (black), ZnO/GCE (red), RGO/GCE (blue), and ZnO/RGO/GCE (green) at a scan rate of 50 mV s^−1^, which proved that the ZnO/RGO/GCE has an enhanced redox reaction with [Fe (CN)_6_]^3−/4−^ than other electrodes. The Nyquist diagrams obtained in 0.1 M KCl containing 2 mM [Fe (CN)_6_]^3−/4−^ during a single experimental session are shown in Fig. 5b. The diagrams span a frequency range from 0.05 Hz to 10 kHz with an amplitude of 5 mV and an applied voltage of 0.22 V. Fig. 5b (inset) displays the fitted Randle's equivalent circuit for the EIS data. The resistance of the solution is indicated by the parameter “*R*_s_”, the charge transfer resistance (“*R*_ct_”), which relates to the charge transfer between the solution and the electrode's surface, the mass diffusion-related Warburg impedance (“*W*”), and the double layer capacitance (“*C*_dl_”) are indicated in this configuration.

The development in the EC kinetics of the redox process is responsible for the values for all electrodes, which are listed in Table 1. It is well known that alterations to the GCE surface can have a substantial effect on the electron transport mechanism of [Fe (CN)_6_]^3−/4−^.

**Table 1:** EIS parameter and its respective values obtained for bare and various modified GCEs

Sample	*R*_s_ (Ω)	*R*_ct_ (Ω)	*C*_dl_ (F)	W (Ω)
Bare GCE	207	178.2	1.42 × 10^−6^	6.25 × 10^−4^
ZnO/GCE	218	1611	8.07 × 10^−7^	5.64 × 10^−4^
RGO/GCE	210	249.8	6.692 × 10^−6^	4.87 × 10^−4^
ZnO/RGO/GCE	209	167.9	2.296 × 10^−5^	5.38 × 10^−4^

The ZnO/RGO/GCE has a higher conductivity and a lower “*R*_ct_” value (167.9 Ω) than the RGO/GCE (249.8 Ω), ZnO/GCE (1611 Ω), and bare GCE (178.2 Ω), according to the CV and EIS data. Based on EIS results, the addition of RGO to the sensing materials has improved the efficiency of electron transport between the electrode and electrolyte, which would increase electrocatalytic activity.

Using the Randles-Sevcik formula (eqn (3)), the electrochemically active surface area (ECSA) of ZnO/RGO/GCE was found to be 0.1193 cm^2^ in 0.1 M KCl containing 2 mM [Fe(CN)_6_]^3−/4−^, which is greater than the ECSA of the bare GCE at 0.0943 cm^2^ (Fig. S3a–d)^67,68^.

3 *I*_p_ = (2.69 × 10^5^)*n*^3/2^*AD*^1/2^*Cν*^1/2^

where *I*_p_ is the oxidation peak current (*A*), n is the number of transferred electrons in the redox reaction (*n* = 1), ‘*A*’ denotes the active surface area of the electrode (cm^2^), *D* denotes the diffusion coefficient (7.6 × 10^−6^ cm^2^ s^−1^), *ν* is the scan rate (V s^−1^), and *C* is the concentration (2 × 10^−6^ mol cm^−3^).

### 3.7. Electrochemical detection of DCF and CMC

**Fig. 6:**
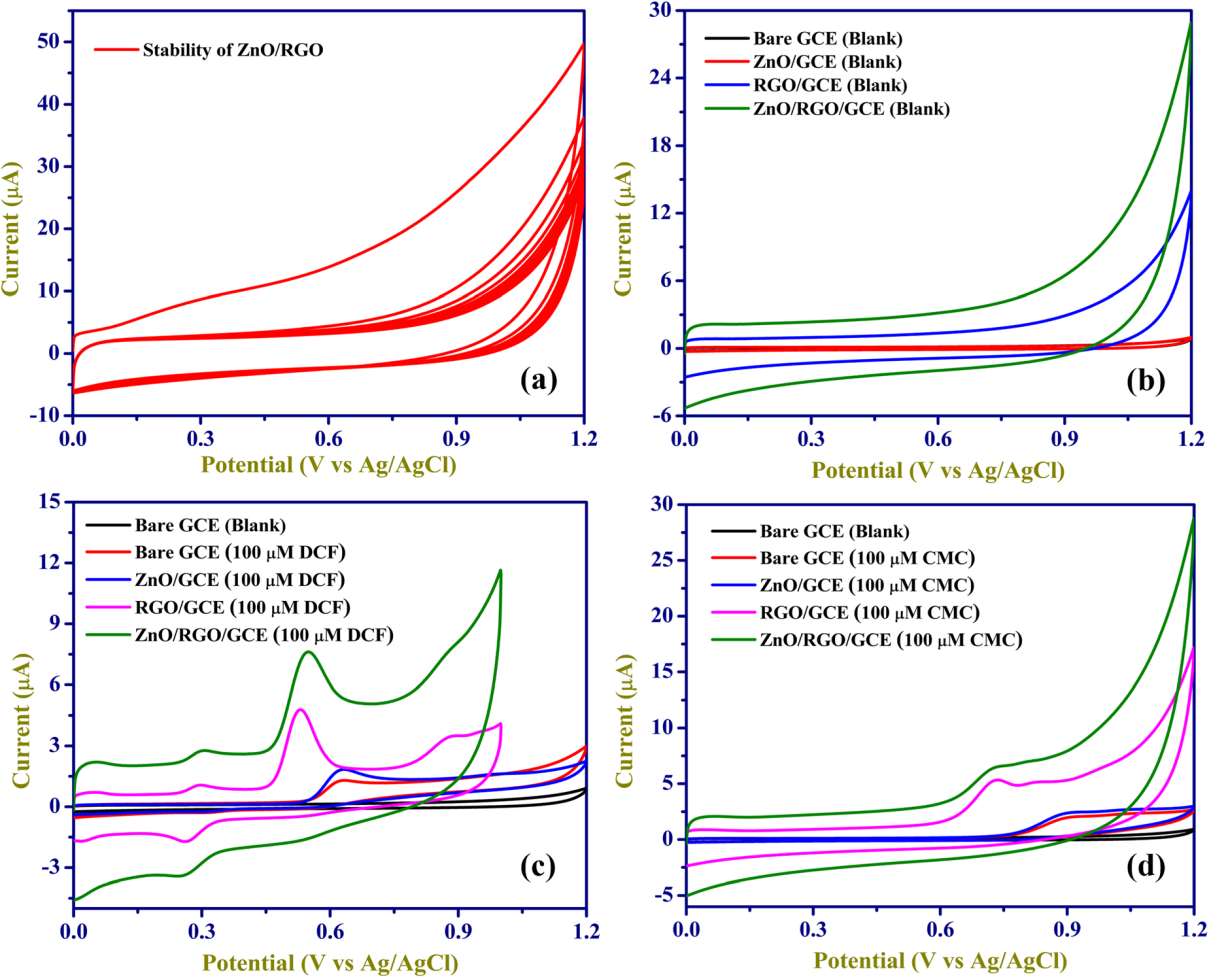
CVs of (a) ZnO/RGO/GCE for 10 cycles. (b) CVs of bare GCE (black), ZnO GCE (red), RGO/GCE (blue), and ZnO/RGO/GCE (green) in 0.1 M PBS blank. CVs of bare GCE blank (black), bare GCE (red), ZnO/GCE (blue), RGO/GCE (pink), and ZnO/RGO/GCE (green) with (c) 100 µM DCF and (d) 100 µM CMC (0.1 M PBS; pH 7.4) at a scan rate of 50 mV s^−1^.

The EC stability of ZnO/RGO/GCE was tested by recording multiple cycles (20 segments) in 0.1 M PBS (pH 7.4) in the potential range of 0 to 1.2 V, as shown in Fig. 6a. The improved electrochemical properties of the ZnO/RGO/GCE were demonstrated in Fig. 6b by comparing them with the ZnO-modified GCE, RGO-modified GCE, and bare GCE. The background current of the ZnO/RGO/GCE was higher than that of the RGO-modified GCE and ZnO-modified GCE. EC oxidation reactions of 100 µM DCF and 100 µM CMC were tested on a bare GCE, ZnO/GCE, RGO/GCE, and ZnO/RGO modified GCE (at a scan rate of 50 mV s^−1^ in 0.1 M PBS, pH 7.4) in the potential range of 0 to 1.2 V.

**Scheme 2:**
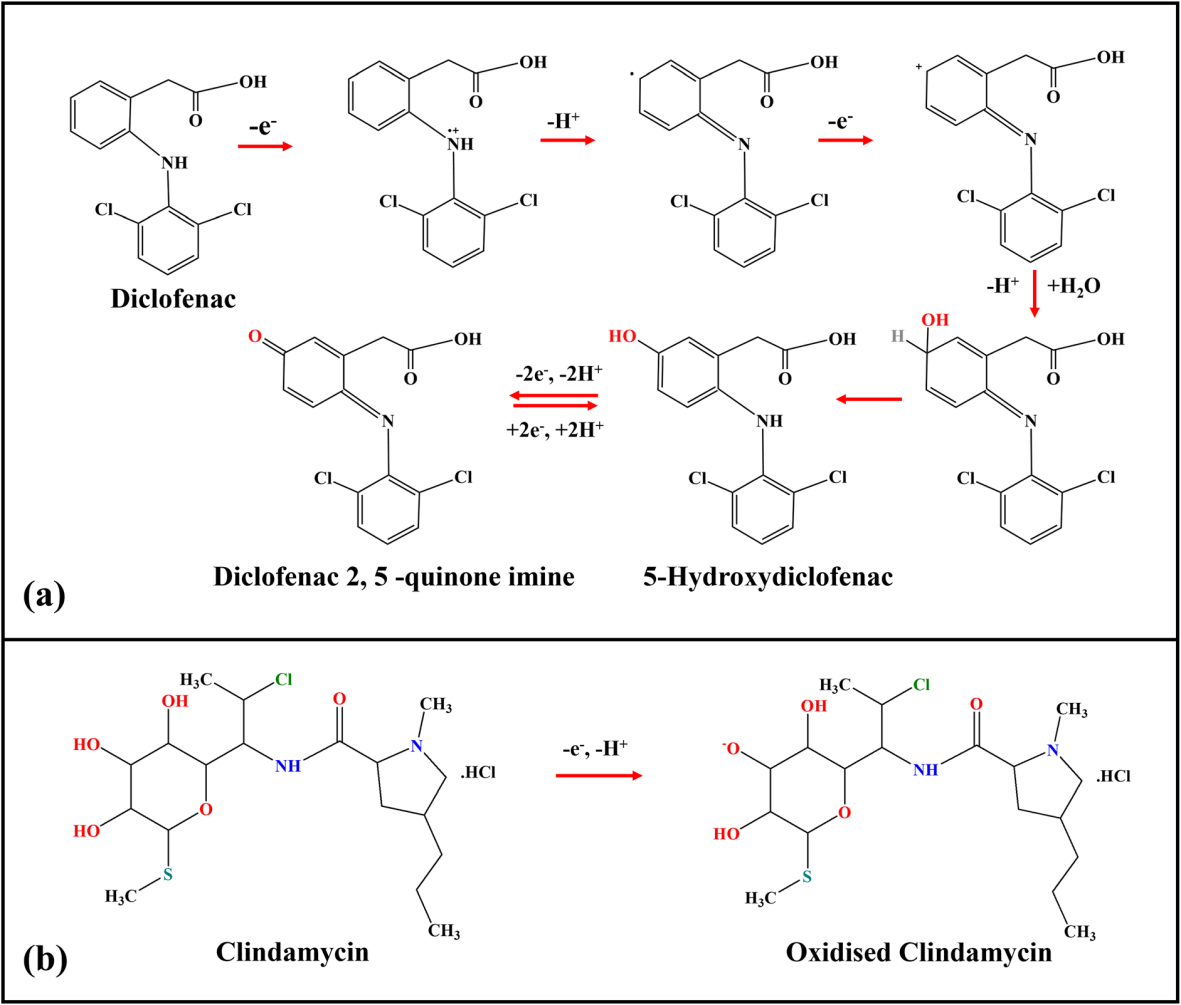
Electrooxidation reactions of DCF (a) and CMC (b).^70,71^

The ZnO/RGO modified GCE exhibited a highly enhanced oxidation peak current at 0.55 V (Fig. 6c, dark green) for DCF and at 0.8 V (Fig. 6d, dark green) for CMC. Whereas DCF and CMC oxidation at the other electrodes of ZnO/GCE and bare GCE exhibit a lesser oxidation peak current at a higher positive peak potential (as shown in Table 2). The modified ZnO/RGO/GCE exhibited a markedly enhanced catalytic current for the oxidation of DCF (5.62-fold increase) and CMC (3.56-fold increase) compared to the bare GCE. It is demonstrated that ZnO/RGO has strong electro-catalytic activity, which is advantageous for DCF and CMC sensing. By comparing with the other electrodes, the ZnO/RGO/GCE showed a lower oxidation potential. For DCF detection, ZnO/RGO/GCE produces a distinct initial anodic current peak at 0.55 V during DCF oxidation, indicating the irreversible formation of 5-hydroxydiclofenac. With repeated scans, the unstable oxidation product of DCF builds up on the electrode surface. During the cathodic cycle, 5-hydroxydiclofenac is reduced to diclofenac 2,5-quinone imine, which is then reoxidized in the following cycle at approximately 0.3 V. The overall EC process involves the transfer of two electrons and two protons, as illustrated in Scheme 2a.

**Table 2:** EC parameters of bare and modified electrodes for electro-oxidation of 100 µM CMC and DCF

S. no.	Electrodes	Oxidation peak current (*I*_pa_) (µA)		Oxidation peak potential (*E*_pa_) (V)	
		DCF	CMC	DCF	CMC
1	Bare GCE	1.36	1.99	0.63	0.90
2	ZnO/GCE	1.86	2.45	0.63	0.90
3	RGO/GCE	4.78	5.31	0.53	0.83
4	ZnO/RGO/GCE	7.65	7.10	0.55	0.80

It had been previously observed by other researchers.^25,69^ The explanation for these peaks can be attributed to the strong adsorption of the reaction products of DCF on the electrode surface, which may be enhanced using ZnO/RGO composite. Also, for the other electrodes, a quasi-reversible reaction was noted. In this study, we observed a one-electron oxidation process of CMC on the ZnO/RGO-modified electrode at approximately 0.80 V (Scheme 2b). The oxidation pathway appeared straightforward and has not been extensively reported in earlier studies. The one-electron behaviour may also reflect the influence of the ZnO/RGO composite, which could enhance the sensitivity and promote a more efficient electron-transfer process. Furthermore, the proposed single-electron mechanism is supported by our scan-rate-dependent EC analysis. These data suggest that modifying the electrode surface with ZnO/RGO improves the electrocatalytic reactions with the DCF and CMC oxidation process.

### 3.8. Effect of scan rate

**Fig. 7:**
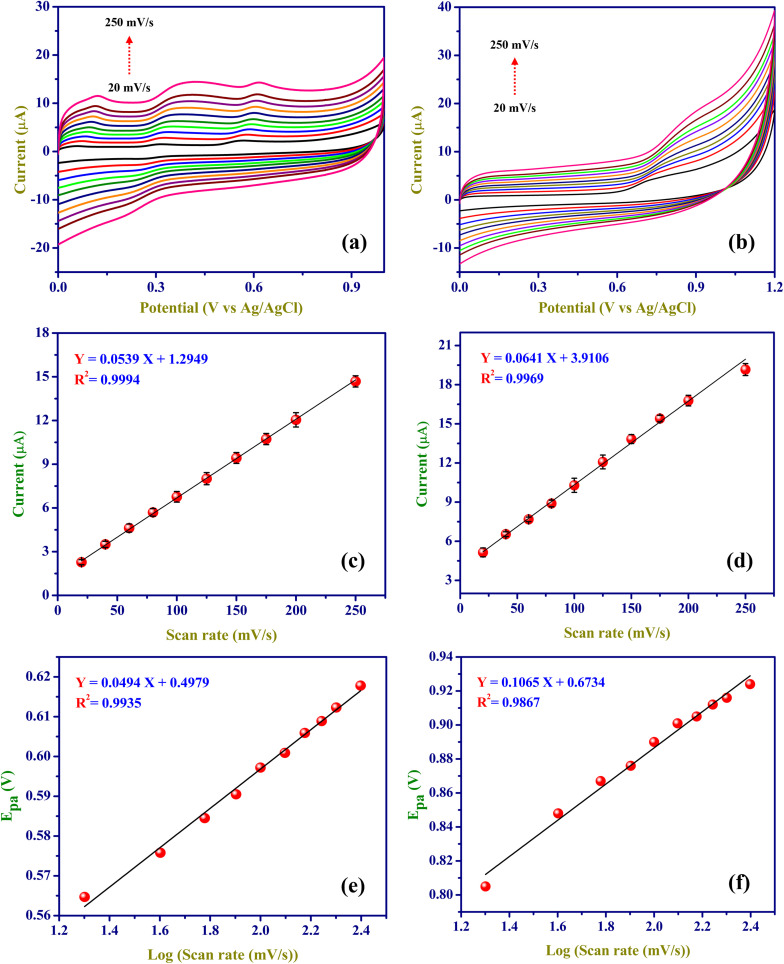
CVs of ZnO/RGO/GCE for electro-oxidations of 50 µM DCF (a), and 200 µM CMC (b) at various scan rates (20 to 250 mV s^−1^; 0.1 M PBS). Linearity graphs were made between the scan rate *vs.* oxidation peak currents (µA) of DCF (c) and CMC (d). Linearity graphs were made for the log of scan rate as a function of *E*_pa_ of DCF (e) and CMC (f).

The impact of the scan rate on the EC oxidation of 50 µM DCF and 200 µM CMC in 0.1 M PBS (pH = 7.4) was investigated. Cyclic voltammograms (CVs) were taken at various scan rates ranging from 20 to 250 mV s^−1^ at ZnO/RGO/GCE, as illustrated in Fig. 7a and b for DCF and CMC, respectively. The linearity of the scan rates for the DCF and CMC to the function of the anodic peak current was plotted. As shown in Fig. 7c and d, the correlation coefficient values indicated that the EC oxidation process is adsorption-controlled.^25^ Linear relationships were observed as follows:

4 *I*_p_ (µA) = 0.0539*ν* (mV s^−1^) + 1.2949(*R*^2^ = 0.9994) (DCF)

5 *I*_p_ (µA) = 0.0641*ν* (mV s^−1^) + 3.9106(*R*^2^ = 0.9969) (CMC)

By applying the slope values obtained from eqn (4)–(6), the surface coverage (*Γ*)^25,72^ of DCF and CMC on ZnO/RGO/GCE was determined to be 1.20 × 10^−10^ mol cm^−2^ and 5.73 × 10^−10^ mol cm^−2^, respectively.6
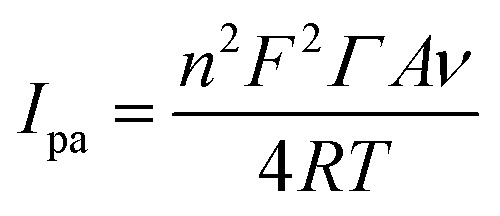


The results depicted that the adsorption of CMC on the ZnO/RGO/GCE surface is slightly more than that of DCF on the electrode. This effective analyte adsorption facilitates the fast oxidation process, thereby improving detection efficiency. Fig. 7e and f illustrate the linearity between the anodic peak potential (*E*_pa_) of DCF and CMC and the log of the scan rate. Linear relationships were observed as follows:

7 *E*_pa_ (V) = 0.0494 log *ν* (mV s^−1^) + 0.4979(*R*^2^ = 0.9935) (DCF)

8 *E*_pa_ (V) = 0.1065 log *ν* (mV s^−1^) + 0.6734(*R*^2^ = 0.9867) (CMC)

Using the slopes from linear eqn (7) and (8) in Laviron's equation^73^ (eqn (9)), it was determined that the electrons (*n*) transferred in the electrooxidation of DCF is 2.39 (approx. *n* = 2), and CMC is 1.11 (approx. *n* = 1). For a fully irreversible reaction, the electron transfer coefficient (*α*) is 0.5.^74^9
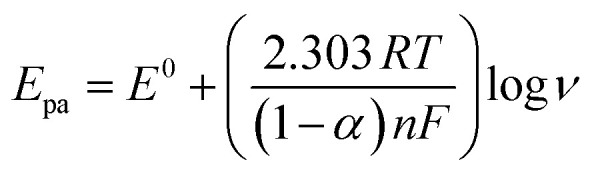
10
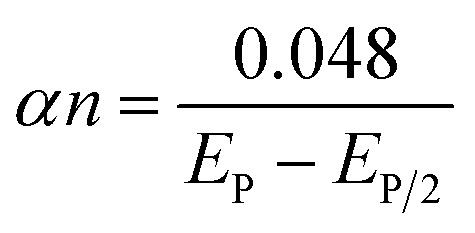
Where *E*_0_ denotes redox potential, *E*_p/2_ represents the potential at the half peak current, *F* (C mol^−1^) is the Faraday constant (96 485 C mol^−1^), and *ν* (V s^−1^) indicates scan rate; other symbols have their standard meanings. Furthermore, the ‘*n*’ value for the electrooxidation of DCF was calculated using the Bard and Faulkner equation^75^ (eqn (10)). The resulting ‘*n*’ value for DCF is 1.87 (*n* = 2), while that for CMC is 0.87 (*n* = 1).

### 3.9. Effect of electrolyte pH on DCF and CMC oxidation

CVs were conducted using a ZnO/RGO/GCE in a series of PBS with different pH values (2, 4, 6, 7.4, and 10).

**Fig. 8:**
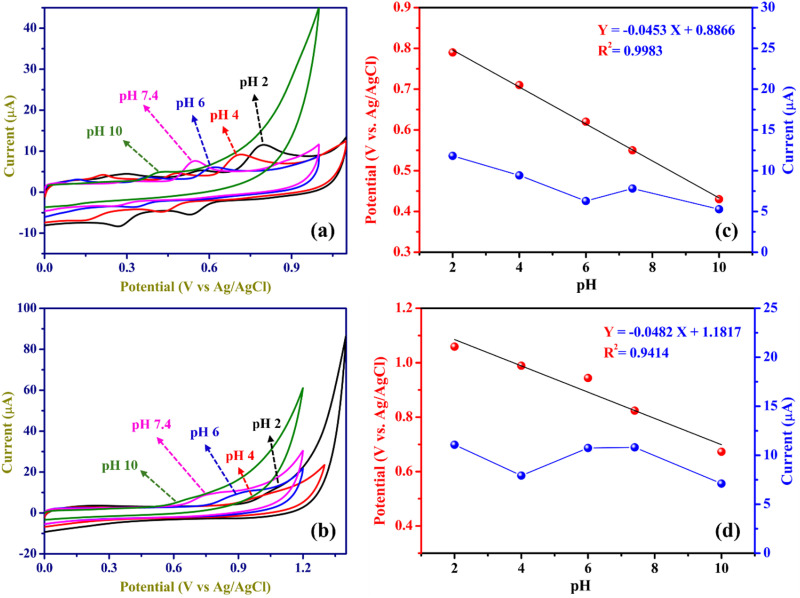
CVs of ZnO/RGO for oxidation of 100 µM DCF (a) and 200 µM CMC (b) in different pH electrolytes. Linear and nonlinear plots were made between the potentials (V *vs.* Ag/AgCl) and oxidation peak currents (µA) *vs.* pH of electrolytes for the oxidation of DCF (c) and CMC (d).

Acetate buffer was used for pH 2, and 0.1 mM HCl in 0.1M KCl was used for pH 4 electrolyte, containing 100 µM DCF and 200 µM CMC. As shown in Fig. 8a and b, the related DCF and CMC oxidation peak currents and peak potentials were measured in various pH electrolytes ranging from pH 2 to 10. The oxidation peak potentials of DCF and CMC shifted to the negative side with an increase in pH, indicating the involvement of protons in the electro-oxidation of DCF and CMC.^27,76^ Additionally, the oxidation peak currents of DCF and CMC were found to be nonlinear with pH. Based on these findings, the ideal pH of the electrolyte was determined to be 7.4 for further studies.11
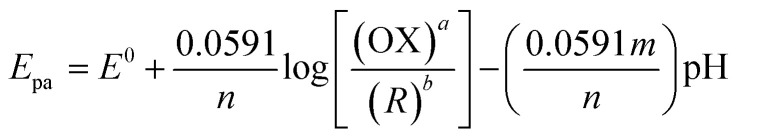


Additionally, Fig. 8c and d illustrate the correlation between oxidation potentials and oxidation peak currents as a function of pH for DCF and CMC, with observed slopes of −0.045 V/pH and −0.048 V/pH, respectively. These values closely approximate the theoretical Nernstian slope of −0.059 V/pH (eqn (11)),^77^ suggesting that the reaction entails an equivalent transfer of protons and electrons.

### 3.10. Amperograms of clindamycin and diclofenac

**Fig. 9:**
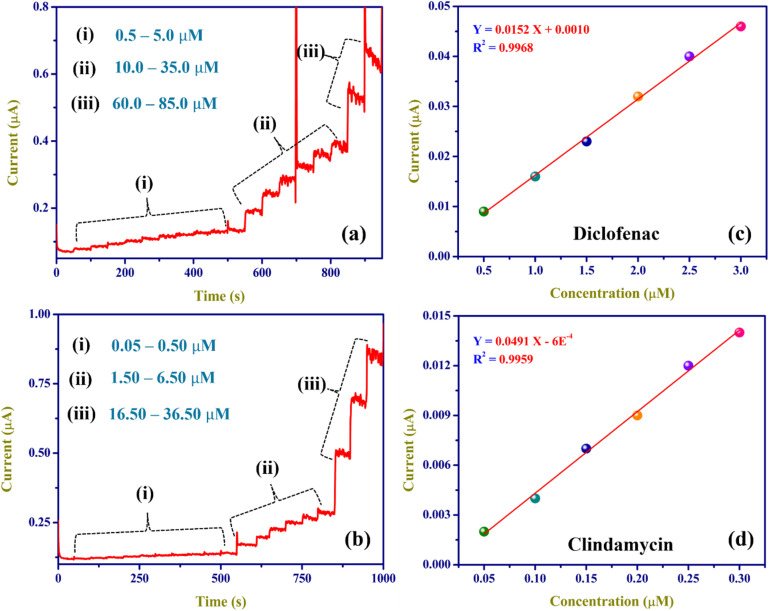
Amperograms were recorded using ZnO/RGO/GCE with DCF at 0.56 V and CMC at 0.8 V, ranging from 0.5 to 85.0 µM (a) and from 0.05 to 36.50 µM (b) at 750 rpm (0.1 M PBS, pH 7.4), respectively. The linear calibration curves were made for a concentration of DCF (0.5 to 85.0 µM) (c) and CMC (0.05 to 36.50 µM) (d) *vs.* oxidation current (µA).

Fig. 9a and b illustrate the amperometric responses of ZnO/RGO/GCE as it oxidises DCF and CMC at various concentrations, using a sensing potential of 0.56 V and 0.8 V, respectively. This experiment was conducted in 0.1 M PBS as the electrolyte, with a pH of 7.4. Following each addition of DCF and CMC, the ZnO/RGO/GCE showed a linear response in current increments. To illustrate the relationship between oxidation peak current and analyte concentration, a calibration plot was made from 0.5 to 3.0 µM for DCF (*R*^2^ = 0.9968) and 0.05 to 0.30 µM for CMC (*R*^2^ = 0.9959) (Fig. 9c and d). The limit of detection (LOD) for DCF and CMC was calculated using the following equation (eqn (12)):12
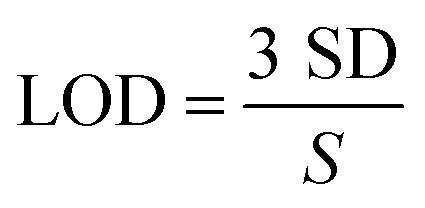


For DCF and CMC, the calibration graph's slope (*S*) was 0.0152 µA µM^−1^ and 0.0491 µA µM^−1^, respectively. The standard deviation of the blank (SD) was 4.041 × 10^−4^ µA and 3 × 10^−4^ µA for DCF and CMC, respectively. Compared to the other techniques discussed, the estimated limits of detection (LODs) were lowest for DCF at 0.079 µM and for CMC at 0.018 µM (Tables 3 and 4), with a lower oxidation potential. Rapid electron movement between the analyte and the electrode surface may be the reason for the enhanced electrocatalytic activity of the hybrid film (ZnO/RGO), as demonstrated by the sensitivity values of 0.127 µA µM^−1^ cm^−2^ for DCF and 0.153 µA µM^−1^ cm^−2^ for CMC (sensitivity = calibration slope/electrode's active surface area). Additionally, the ZnO/RGO's larger electro-active surface area might have created a bulk porosity matrix with more active sites, which is advantageous for the DCF and CMC oxidation reactions.

**Table 3:** Comparison of DCF detection with other previously reported electrodes^a^

Electrode	pH	Method	Linear range (µM)	LOD (µM)	Reference
PDP graphite	7	DPV	8.19–111	2.45	71
PSP graphite	7	DPV	2.56–9.5	0.76	71
Graphite/Co_1.2_Fe_1.8_O_4_	7	DPV	0.01–23.1	0.001	24
GO/PHEN	7	CV	0.01–1.75	0.0015	25
PDDA-graphene	7	DPV	10–100	0.609	78
MWCNT/CPE	8.0	DPV	0.1–90	0.74	79
MWCNTs-vinyl ferrocene/CPE	7.0	SWV	5.0–600.0	2	80
ZnO/RGO	7.4	Amperometry	0.5 to 85	0.079	Our work

a PDP – Potentiodynamic pretreatment, PSP – Potentiodynamic pretreatment, Co_1.2_Fe_1.8_O_4_ – Cobalt ferrite, PHEN – Phenanthroline, PDDA – poly (diallyl dimethylammonium chloride), CPE – Carbon paste electrode, MWCNT – Multiwall carbon nanotubes.

**Table 4:** Comparison of CMC detection with other previously reported electrodes^a^

Electrode	pH	Method	Linear range (µM)	LOD (µM)	Reference
GO/Au/chi/GCE	7	DPV	0.95–140	0.29	81
CPE	10	DPV	0.2–1.0	0.077	71
Au@Ag NPs	5.02	Colorimetric	0.65–7.5	0.2	82
TR	7.5	CE-ECL	0.5–100	0.14	18
EP-EPPG	7	CV, DPV	0.2–16.0, 0.03–3.2	0.06, 0.01	83
FFASV (microgold electrode)	8.0	SWV	9.4–100	3	84
Zn–Al (LDH)	3.6	CV	4–700	0.044	27
ZnO/RGO	7.4	i-t	0.05 to 36.50	0.018	Our work

a GO/Au/chi/GCE – Graphene oxide/Gold nanoparticles/chitosan/glassy carbon electrode, FFASV – Fast Fourier adsorptive stripping voltammetry, TR – tris(2,2′-bipyridine) ruthenium(ii), CE-ECL – Coupled capillary electrophoresis with end-column electrogenerated chemiluminescence, CV – cyclic voltammetry, DPV–differential pulse voltammetry, SWV – square wave voltammetry, *i*–*t*–amperometry, Zn – Al (LDH) – zinc aluminium (layer double hydroxide).

### 3.11. Interference, reproducibility and stability of DCF and CMC sensors

To evaluate the sensor's performance in a real-world sample, amperometric measurements were conducted to determine the selectivity of a ZnO/RGO-modified electrode against various common ions and molecules. Interferents such as ammonium chloride (NH_4_Cl), magnesium chloride (MgCl_2_), sodium sulphate (Na_2_SO_4_), zinc acetate (Zn (CH_3_COO)_2_), potassium chloride (KCl), sodium chloride (NaCl), urea, dextrose (DEX), sulfamethazine (SMZ), and chloramphenicol (CPCL) were added at concentrations of tenfold higher than those of the analytes. These compounds were introduced into the amperogram in 0.1 M PBS (pH 7.4), containing 5 µM DCF and 20 µM CMC. Human urine contains various ions and molecules, including antibiotics such as CPCL and SMZ, which were selected to test selectivity. Other common molecules, like dopamine and ascorbic acid, were excluded due to their low concentrations and negligible impact on DCF and CMC detection. The oxidation peak current values for DCF (Fig. S4a) and CMC (Fig. S4b) were not changed significantly after adding interferents, indicating the ZnO/RGO NCs modified electrode is selective for DCF and CMC. Thus, this electrode is suitable for detecting DCF and CMC in biological samples. The sensor's reproducibility was tested by preparing the electrode three separate times and evaluating its response to 100 µM DCF (green) and 200 µM CMC (red) oxidation. The results showed no significant variation in the oxidation peak current values, indicating that the sensor exhibited reliable reproducibility (Fig. S4c). Additionally, from Fig. S4d, it is shown that the stability of the ZnO/RGO sensor for the amperometric detection of 5 µM DCF and 20 µM CMC is only reduced by 8.16% and 15.44% after 500 seconds of amperometric run in the continuous stirring of 750 rpm.

### 3.12. Real sample analysis

The ZnO/RGO/GCE was used in pH 7.4, 0.1 M PBS for amperometric measurements with human urine samples spiked with DCF and CMC standards. After adding 1 mM standard solutions of DCF and CMC to urine, concentrations were measured using the same sensor. Experiments included separate additions of 0.50, 1.00, and 1.50 µM DCF, and 0.50, 1.00, and 1.50 µM CMC in PBS. The standard addition method determined DCF and CMC levels in urine samples. As shown in Table 5, DCF recoveries ranged from 90.0% to 93.0%, and CMC from 99.3% to 106.0%. These findings demonstrated that the ZnO/RGO/GCE sensor can accurately determine DCF and CMC in a urine sample.

**Table 5:** Real sample analysis in a human urine sample

Sample	Added (µM)		Found (µM)			Recovery (%)
	DCF	CMC	DCF	CMC	DCF	CMC
Human urine	0.50	0.50	0.45	0.53	90.0	106.0
	1.00	1.00	0.93	1.00	93.0	100.0
	1.50	1.50	1.35	1.49	90.0	99.3

## 4. Conclusion

In this study, GO was successfully synthesised using the modified Hummers' method, and ZnO was synthesised using the hydrothermal method. These were then combined using the sonication method and electrochemically reduced for the detection of antibiotics CMC and DCF. Various tests, such as UV-vis, FTIR, XRD, HR-SEM, EDAX, TGA, DLS, Zeta potential and EIS, confirmed the successful synthesis of the ZnO/GO composite. Compared to the bare GCE, ZnO/GCE, and RGO/GCE, the ZnO/RGO/GCE showed an excellent response to CMC and DCF detection, with a higher peak current. Tests with different concentrations, scan rates, and various electrolyte pH levels were conducted, revealing a linear range of determinations from 0.5 to 85.0 µM for DCF and 0.05 to 36.50 µM for CMC. The LOD were calculated to be 0.079 µM for DCF and 0.018 µM for CMC by amperometry. The proposed sensor was also applied in a urine sample, which showed DCF recoveries ranged from 90.0% to 93.0%, and CMC from 99.3% to 106.0%. Overall, this study demonstrated that the ZnO/RGO composite material is a selective and promising electrocatalyst for the detection of CMC and DCF. It can be useful for the development of a point-of-care tool for the detection of CMC and DCF in real-world samples.

## Author contributions

Gokul Sridharan: writing – original draft. Gokul Sridharan, Surendar Balu, and Raji Atchudan: methodology, formal analysis, conceptualisation, writing – original draft. Dhanraj Ganapathy: validation, data curation. Chandramohan Govindasamy: resources, review and validation. Sandeep Arya: editing and validation, formal analysis. Ashok K. Sundramoorthy: writing – review & editing, funding acquisition, supervision, project administration.

## Conflicts of interest

The authors declare that they have no known competing financial interests or personal relationships that could have appeared to influence the work reported in this paper.

## Supplementary Material

RA-015-D5RA07011B-s001

## Data Availability

All the experimental data obtained in this work are presented in the manuscript itself, and no additional datasets were excluded. Supplementary information (SI): material characterisation – thermogravimetric analysis (TGA), dynamic light scattering (DLS), and zeta potential of the ZnO/RGO nanocomposite. electrochemical studies – different scan rate effect for bare GCE and ZnO/RGO/GCE in 2 mM [Fe (CN)_6_]^3−/4−^ and linear plot for the sqrt of the scan rate *vs.* anodic peak current. Interference, reproducibility and stability of the ZnO/RGO/GCE for the detection of DCF and CMC. See DOI: https://doi.org/10.1039/d5ra07011b.

## References

[cit1] Meti M. D., Nandibewoor S. T., Chimatadar S. A. (2014). Monatsh. Chem..

[cit2] Baudet M. (1976). J. Pharm. Belg..

[cit3] Murphy P. B. , Bistas K. G., Patel P. and Le J. K., in StatPearls, StatPearls Publishing, Treasure Island (FL), 2025

[cit4] Bouazza N., Pestre V., Jullien V., Curis E., Urien S., Salmon D., Tréluyer J.-M. (2012). Br. J. Clin. Pharmacol..

[cit5] Urabe A., Takaku F., Mizoguchi H., Nomura T., Aoki N., Yamaguchi H., Mutoh Y., Miura Y., Toyama K., Hirashima K. (1990). Jpn. J. Antibiot..

[cit6] Davey M. G., Birrane J., Brennan M., Breen D. P., Laing M. E. (2020). Oxford Med. Case Rep..

[cit7] Altman R., Bosch B., Brune K., Patrignani P., Young C. (2015). Drugs.

[cit8] McGettigan P., Henry D. (2013). PLoS Med..

[cit9] Singh R., Bansal D., Baduni N., Vajifdar H. (2011). Indian J. Crit. Care Med..

[cit10] Michel M. C., Staskin D. (2022). Biomedicines.

[cit11] Treatment, https://www.who.int/teams/control-of-neglected-tropical-diseases/leprosy/treatment, accessed November 1, 2025

[cit12] Ervens J., Schiffmann L., Berger G., Hoffmeister B. (2004). J. Craniomaxillofac. Surg..

[cit13] Sridharan G., Keskar V., Ganapathy D., Atchudan R., Arya S., Sundramoorthy A. K. (2025). ECS J. Solid State Sci. Technol..

[cit14] Sarfraz S., Hussain S., Javed M., Raza A., Iqbal S., Alrbyawi H., Aljazzar S. O., Elkaeed E. B., Somaily H. H., Pashameah R. A., Alzahrani E., Farouk A.-E. (2022). Inorganics.

[cit15] González L., Yuln G., Volonté M. G. (1999). J. Pharm. Biomed. Anal..

[cit16] Barazandeh Tehrani M., Namadchian M., Fadaye Vatan S., Souri E. (2013). Daru.

[cit17] Botello J. C., Pérez-Caballero G. (1995). Talanta.

[cit18] Wang J., Peng Z., Yang J., Wang X., Yang N. (2008). Talanta.

[cit19] Jin W., Zhang J. (2000). J. Chromatogr. A.

[cit20] Wei G., Dang G., Li H. (2007). Luminescence.

[cit21] Sridharan G., Atchudan R., Magesh V., Arya S., Ganapathy D., Nallaswamy D., Sundramoorthy A. K. (2023). Electroanalysis.

[cit22] Sidhu M. S., Sridharan G., Ganapathy D., Atchudan R., Arya S., Mahadevegowda S. H., Sundramoorthy A. K. (2025). RSC Adv..

[cit23] Subash R., Sridharan G., Nallaswamy D., Atchudan R., Arya S., Sundramoorthy A. K. (2024). Nanosci. Nanotechnol. – Asia.

[cit24] Sundaresan P., Lee T. Y. (2022). Microchem. J..

[cit25] Măghinici A.-R., Bounegru A.-V., Apetrei C. (2025). Chemosensors.

[cit26] El-Wekil M. M., Alkahtani S. A., Ali H. R. H., Mahmoud A. M. (2018). J. Mol. Liq..

[cit27] Ahmed A. M. S., Radalla A. M., Mahgoub S. M., Elsuccary S. A. A., Korany M. A., Allah A. E., Mohamed F., Allam A. A., Alfassam H. E., Mahmoud R. (2025). Fenxi Huaxue.

[cit28] Kumaresan L., Palanisamy G., Lee J. (2024). J. Mater. Chem. C.

[cit29] Kala K., Padmasini N., Harish M. N., Shanmuga priyan J., Siranjeevi R. (2024). Surf. Interfaces.

[cit30] Indumathi N., Sridevi C., Madona J., Gokulavani G., Prabhu S. (2025). Inorg. Chem. Commun..

[cit31] Zhang R., Pang H. (2021). J. Energy Storage.

[cit32] Costa M. C. F., Marangoni V. S., Ng P. R., Nguyen H. T. L., Carvalho A., Castro Neto A. H. (2021). Nanomaterials.

[cit33] Shahdeo D. , Roberts A., Abbineni N. and Gandhi S., in Comprehensive Analytical Chemistry, Elsevier, 2020, pp. 175–199

[cit34] Kumar A., Srivastava S., Bosetti P., Kumar V., Tiwari D. K., Mishra D. P., Chandel V. S., Srivastava S. (2025). Diam. Relat. Mater..

[cit35] Singh A. K. (2010). Adv. Powder Technol..

[cit36] Maafa I. M. (2025). Biosensors.

[cit37] Raj M. A. and John S. A., in Graphene-Based Electrochemical Sensors for Biomolecules, Elsevier, 2019, pp. 1–41

[cit38] Salih E., Mekawy M., Hassan R. Y. A., El-Sherbiny I. M. (2016). J. Nanostruct. Chem..

[cit39] Poomporai Vadivel R., Venkatesh K., Alagarsamy S., Albeshr M. F., Krishnapandi A., Sivaganesh D., Arulanandam X., Ramaraj S. K. (2024). J. Electrochem. Soc..

[cit40] Jain B., Hashmi A., Sanwaria S., Singh A. K., Susan M. A. B. H., Singh A. (2020). Adv. Compos. Hybrid Mater..

[cit41] Raizada P., Sudhaik A., Singh P. (2019). Mater. Sci. Energy Technol..

[cit42] Singh M., Sridharan G., Ganapathy D., Sundramoorthy A. K. (2025). Nanosci. Nanotechnol. – Asia.

[cit43] Vekhande H. N., Bagawade J. A. (2025). Fullerenes, Nanotub. Carbon Nanostruct..

[cit44] Zaaba N. I., Foo K. L., Hashim U., Tan S. J., Liu W.-W., Voon C. H. (2017). Procedia
Eng..

[cit45] Gurunathan S., Han J. W., Dayem A. A., Eppakayala V., Kim J.-H. (2012). Int. J. Nanomed..

[cit46] Lin Y., Hong R., Chen H., Zhang D., Xu J. (2020). J. Nanomater..

[cit47] Djafarou R., Brahmia O., Haya S., Sahmetlioglu E., Kılıç Dokan F., Hidouri T. (2025). Int. J. Mol. Sci..

[cit48] Prabhakaran P. K., Balu S., Sridharan G., Ganapathy D., Sundramoorthy A. K. (2025). Eng. Res. Express.

[cit49] Thamilselvan V., Balu S., Ganapathy D., Atchudan R., Arya S., Hazra S., Sundramoorthy A. K. (2025). Results Surf. Interfaces.

[cit50] Inagaki M. and Kang F., in Materials Science and Engineering of Carbon: Fundamentals, Elsevier, 2014, pp. 219–525

[cit51] Davis K., Yarbrough R., Froeschle M., White J., Rathnayake H. (2019). RSC Adv..

[cit52] Saranya M., Ramachandran R., Wang F. (2016). J. Sci. Adv. Mater. Devices.

[cit53] Mututu V., Sunitha A. K., Thomas R., Pandey M., Manoj B. (2019). Int. J. Electrochem. Sci..

[cit54] Shen L., Zhang L., Wang K., Miao L., Lan Q., Jiang K., Lu H., Li M., Li Y., Shen B., Zheng W. (2018). RSC Adv..

[cit55] Saha J. K., Podder J. (1970). J. Bangladesh Acad. Sci..

[cit56] Kalpana V. N., Kataru B. A. S., Sravani N., Vigneshwari T., Panneerselvam A., Devi Rajeswari V. (2018). OpenNano.

[cit57] Sridharan G., Babu K. L., Ganapathy D., Atchudan R., Arya S., Sundramoorthy A. K. (2023). Crystals.

[cit58] Prabhu Y. T., Rao K. V., Kumar V. S. S., Kumari B. S. (2014). World J. Nano Sci. Eng..

[cit59] Sridharan G., Godwin C. J. T., Atchudan R., Arya S., Govindasamy M., Osman S. M., Sundramoorthy A. K. (2024). J. Taiwan Inst. Chem. Eng..

[cit60] Sridharan G., Murugan R. V., Atchudan R., Arya S., Sundramoorthy A. K. (2025). Nano Life.

[cit61] Murugan R. V., Sridharan G., Atchudan R., Arya S., Nallaswamy D., Sundramoorthy A. (2024). Curr. Nanosci..

[cit62] Zhou W. D., Wu X., Zhang Y. C., Zhang M. (2007). Mater. Lett..

[cit63] Bekele E. A., Korsa H. A., Desalegn Y. M., Bekele G. G. (2025). J. Alloys Compd..

[cit64] Shukla P., Shukla J. K. (2019). Phys. B Condens. Matter.

[cit65] Zhang F., Lan J., Yang Y., Wei T., Tan R., Song W. (2013). J. Nanopart. Res..

[cit66] Lazanas A. C., Prodromidis M. I. (2023). ACS Meas. Sci. Au.

[cit67] Pang Y.-H., Huang Y.-Y., Wang L., Shen X.-F., Wang Y.-Y. (2020). Environ. Pollut..

[cit68] Gissawong N., Srijaranai S., Boonchiangma S., Uppachai P., Seehamart K., Jantrasee S., Moore E., Mukdasai S. (2021). Mikrochim. Acta.

[cit69] Goyal R. N., Chatterjee S., Agrawal B. (2010). Sens. Actuators, B.

[cit70] Cid-Cerón M. M., Guzmán-Hernández D. S., Ramírez-Silva M. T., Galano A., Romero-Romo M., Palomar-Pardavé M. (2016). Electrochim. Acta.

[cit71] Aguilar-Lira G. Y., Álvarez-Romero G. A., Zamora-Suárez A., Palomar-Pardavé M., Rojas-Hernández A., Rodríguez-Ávila J. A., Páez-Hernández M. E. (2017). J. Electroanal. Chem..

[cit72] Bounegru A. V., Apetrei C. (2022). Int. J. Mol. Sci..

[cit73] Laviron E. (1979). J. Electroanal. Chem. Interfacial Electrochem..

[cit74] Blel A., García-Guzmán J. J., Cubillana-Aguilera L., Palacios-Santander J. M., Dridi C. (2025). RSC Adv..

[cit75] bard A. J. and faulkner L. R., in Electrochemical Methods: Fundamentals and Applications, Springer Science and Business Media LLC, New York, Wiley, 2001, 2nd edn, 2002, vol. 38, p. 236

[cit76] Lochab A., Baweja S., Jindal K., Chowdhuri A., Tomar M., Saxena R. (2025). Microchem. J..

[cit77] Rajendran J., Reshetilov A. N., Sundramoorthy A. K. (2021). Mater. Adv..

[cit78] Okoth O. K., Yan K., Liu L., Zhang J. (2016). Electroanalysis.

[cit79] Aguilar-Lira G. Y., Gutiérrez-Salgado J. M., Rojas-Hernández A., Rodríguez-Ávila J. A., Páez-Hernández M. E., Álvarez-Romero G. A. (2018). ECS Trans..

[cit80] Mokhtari A., Karimi-Maleh H., Ensafi A. A., Beitollahi H. (2012). Sens. Actuators, B.

[cit81] Wong A., Razzino C. A., Silva T. A., Fatibello-Filho O. (2016). Sens. Actuators, B.

[cit82] Hajjizadeh M., Jabbari A., Heli H., Moosavi-Movahedi A. A., Haghgoo S. (2007). Electrochim. Acta.

[cit83] Hadi M., Honarmand E. (2017). Russ. J. Electrochem..

[cit84] Ganjali M. R., Faridbod F., Nasli-Esfahani E., Larijani B., Rashedi H., Norouzi P. (2010). Int. J. Electrochem. Sci..

