# Supplementary material for: Electrochemical detection of diclofenac and clindamycin using ZnO nanorods/RGO nanocomposite modified electrode
Source: RSC Adv. 2025 Dec 10;15(57):49094–108. doi: 10.1039/d5ra07011b (PMC12691350; doi:10.1039/d5ra07011b)
Supplement: RA-015-D5RA07011B-s001 [file RA-015-D5RA07011B-s001.pdf]

Supplementary Information (SI) for RSC Advances.
This journal is © The Royal Society of Chemistry 2025

SUPPLEMENTARY INFORMATION

Electrochemical Detection of Clindamycin and Diclofenac Using ZnO Nanorods/RGO Nanocomposite Modified Electrode

Gokul Sridharan[1,#], Surendar Balu[1#], Raji Atchudan[2#], Chandramohan Govindasamy[3], Dhanraj Ganapathy[1], Sandeep Arya[4], and Ashok K. Sundramoorthy[1,*]

[1]*Centre for Nano-Biosensors, Department of Prosthodontics, Saveetha Dental College and Hospitals, Saveetha Institute of Medical and Technical Sciences, Poonamallee High Road, Velappanchavadi, Chennai, 600077, Tamil Nadu, India*

[2]*School of Chemical Engineering, Yeungnam University, Gyeongsan 38541, Republic of Korea*

[3]*Department of Community Health Sciences, College of Applied Medical Sciences, King Saud University, P.O. Box 10219, Riyadh 11433, Saudi Arabia.*

[4]*Department of Physics, University of Jammu, Jammu, Jammu and Kashmir 180006, India*

*Corresponding author:

Email: ashok.sundramoorthy@gmail.com

#These authors contributed equally.

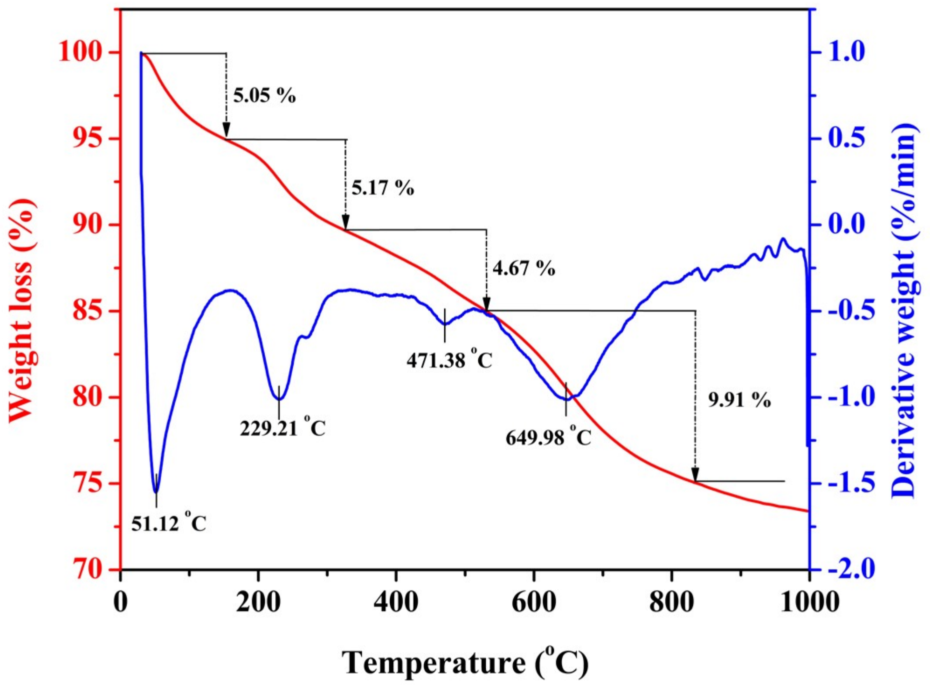

Figure S1: TGA curve of ZnO/GO nanocomposite.

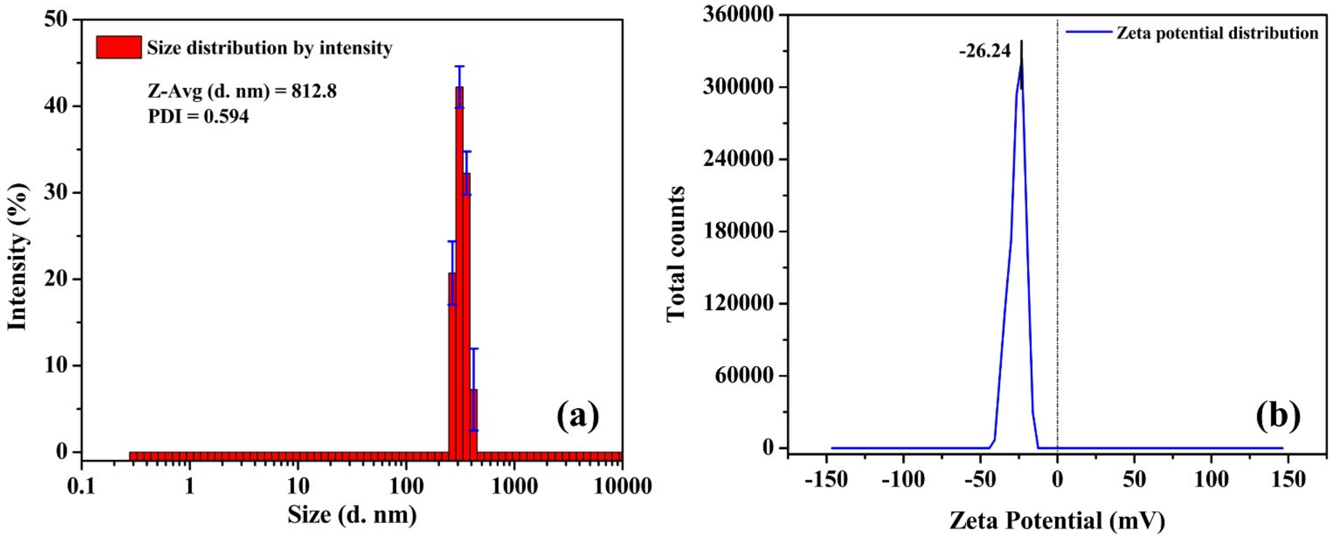

Figure S2: DLS (a) and Zeta potential (b) of ZnO/GO nanocomposite dispersion.

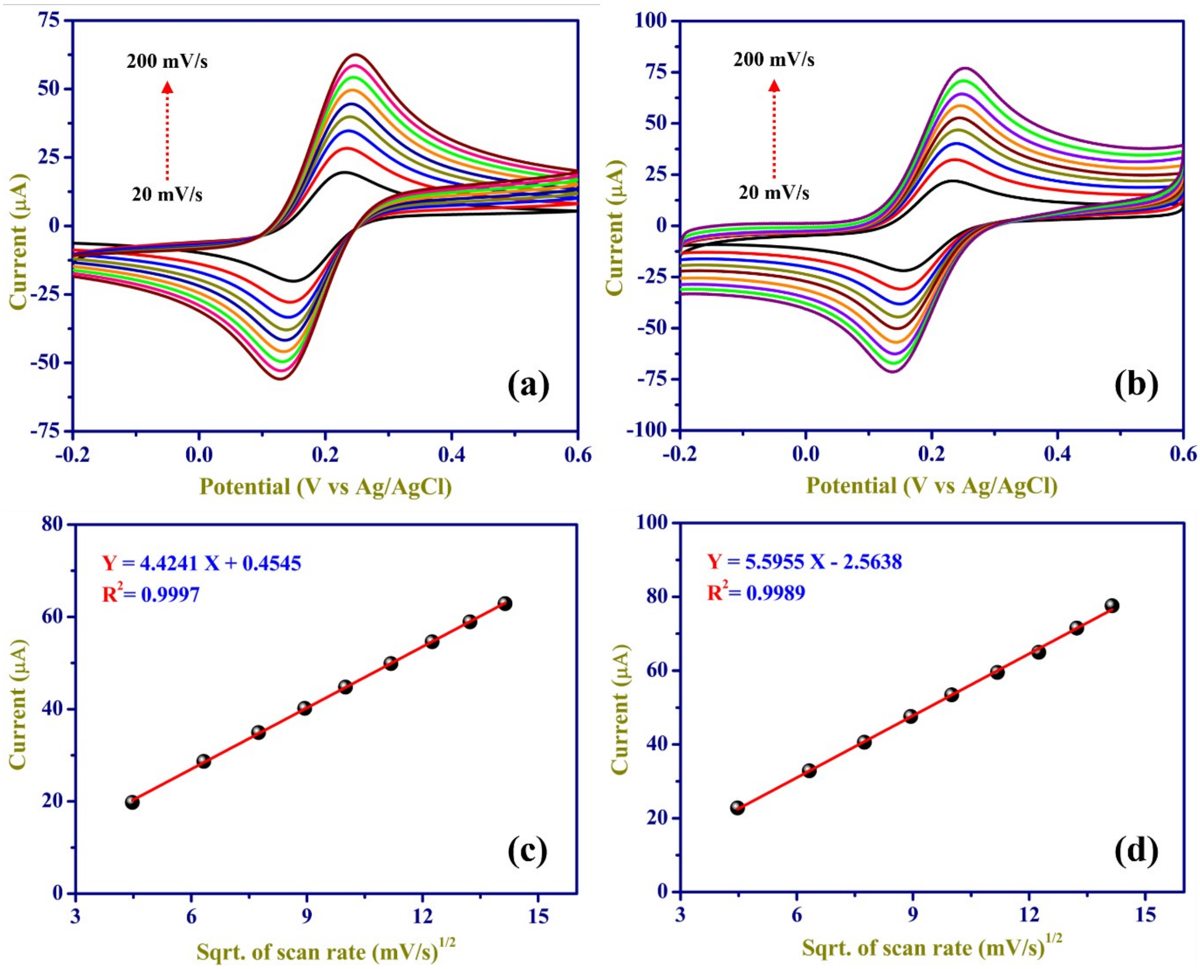

Figure S3: Cyclic voltammograms of bare GCE (a) and ZnO/RGO/GCE (b) in 0.1 M KCl containing 2 mM $[Fe(CN)_6]^{-3/-4}$ at a different scan rate (20, 40, 60, 80, 100, 125, 150, 175, 200 mV/s). Linear plots were drawn between the anodic peak current of $[Fe(CN)_6]^{-3/-4}$ and the square root of the scan rate (mV/s) on bare GCE (c) and ZnO/RGO/GCE (d).

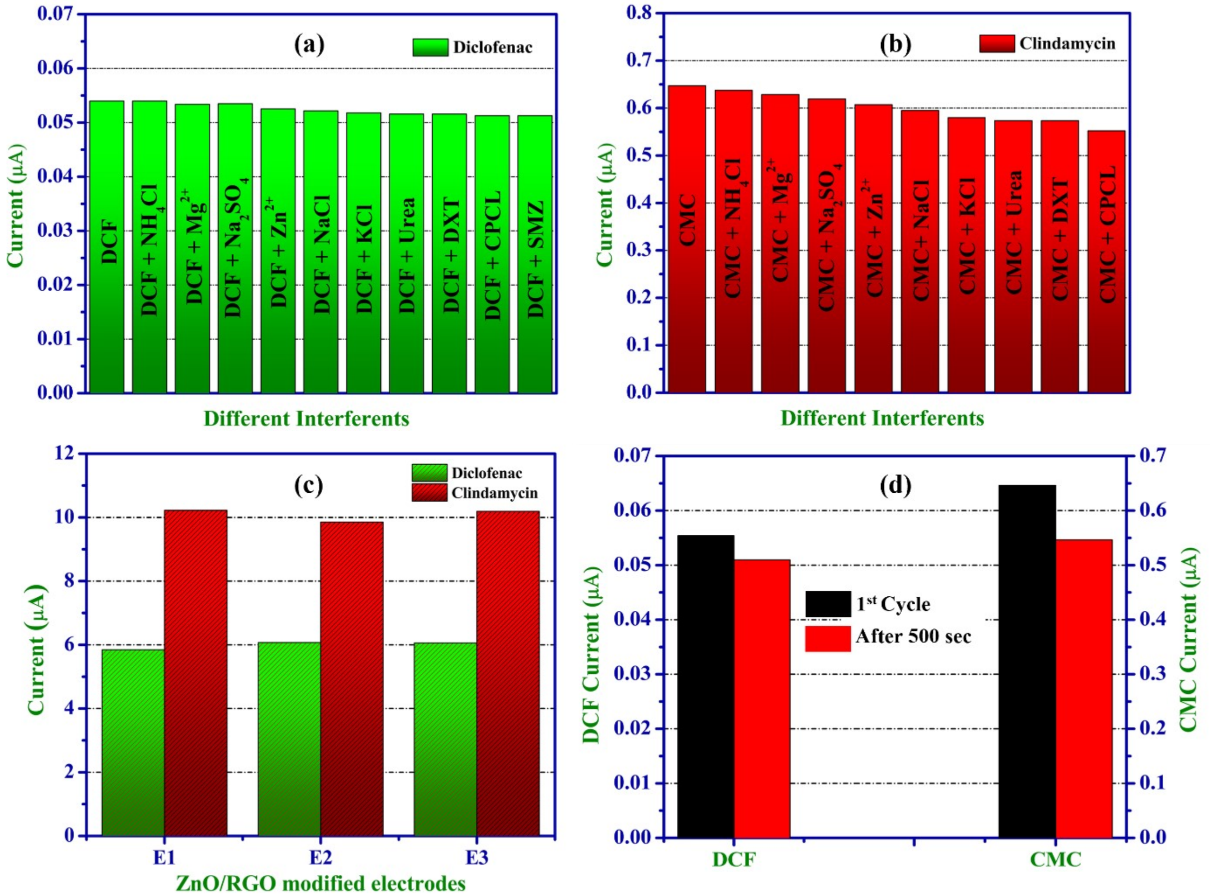

Figure S4: Interference studies were carried out. The bar graphs depict the effect of interference with (a) 5 μM DCF and (b) 20 μM CMC. (c) The bar graph illustrates the reproducibility of the ZnO/RGO-modified sensor for detecting 100 μM DCF and 200 μM CMC. (d) The bar graph illustrates the stable response data of the ZnO/RGO/GCE for the detection of 5 μM DCF and 20 μM CMC. We have compared the oxidation peak currents (μA) of the 1st cycle (black) and after 500 sec (red).
